# Honey can inhibit and eliminate biofilms produced by *Pseudomonas aeruginosa*

**DOI:** 10.1038/s41598-019-54576-2

**Published:** 2019-12-03

**Authors:** Jing Lu, Nural N. Cokcetin, Catherine M. Burke, Lynne Turnbull, Michael Liu, Dee A. Carter, Cynthia B. Whitchurch, Elizabeth J. Harry

**Affiliations:** 1 The ithree institute, University of Technology Sydney, Ultimo, NSW 2007 Australia; 2 School of Life and Environmental Sciences, University of Sydney, Sydney, NSW 2006 Australia

**Keywords:** Antimicrobial resistance, Antimicrobial resistance, Biofilms, Biofilms

## Abstract

Chronic wound treatment is becoming increasingly difficult and costly, further exacerbated when wounds become infected. Bacterial biofilms cause most chronic wound infections and are notoriously resistant to antibiotic treatments. The need for new approaches to combat polymicrobial biofilms in chronic wounds combined with the growing antimicrobial resistance crisis means that honey is being revisited as a treatment option due to its broad-spectrum antimicrobial activity and low propensity for bacterial resistance. We assessed four well-characterised New Zealand honeys, quantified for their key antibacterial components, methylglyoxal, hydrogen peroxide and sugar, for their capacity to prevent and eradicate biofilms produced by the common wound pathogen *Pseudomonas aeruginosa*. We demonstrate that: (1) honey used at substantially lower concentrations compared to those found in honey-based wound dressings inhibited *P. aeruginosa* biofilm formation and significantly reduced established biofilms; (2) the anti-biofilm effect of honey was largely driven by its sugar component; (3) cells recovered from biofilms treated with sub-inhibitory honey concentrations had slightly increased tolerance to honey; and (4) honey used at clinically obtainable concentrations completely eradicated established *P. aeruginosa* biofilms. These results, together with their broad antimicrobial spectrum, demonstrate that manuka honey-based wound dressings are a promising treatment for infected chronic wounds, including those with *P. aeruginosa* biofilms.

## Introduction

The management and treatment of chronic wounds is an increasingly difficult and costly problem, further exacerbated when the wounds become infected^1^. Bacterial biofilms, where cells are embedded within a matrix comprised of exopolysaccharides and other components including DNA, proteins, and membrane vesicles, are the major cause of chronic wound infections and are notoriously resistant to treatment with antibiotics^2,3^.

*Pseudomonas aeruginosa* is a particularly virulent wound pathogen and is commonly isolated from the polymicrobial biofilms found in chronic wounds^4–7^. Infections caused by *P. aeruginosa* are especially difficult to treat due to the inherent antibiotic resistance mechanisms possessed by the organism. This includes multi-drug efflux pumps that remove antibiotics from inside the cell before they can act on specific targets^8–10^. Additionally, the physical structure of the extracellular *P. aeruginosa* biofilm matrix inhibits penetration of the biofilm, and so *P. aeruginosa* associated chronic wound infections often do not respond to treatments with conventional antibiotics^11,12^. The need for new approaches to combat polymicrobial biofilms in chronic wounds (particularly those colonised by *P. aeruginosa*), combined with the current and growing crisis of antimicrobial resistance warrants investigation into the use of complex natural products with antimicrobial activity as potential treatment avenues.

Prior to the introduction of modern antibiotics, treating wounds with honey was a common and effective practice, almost certainly due to its potent antimicrobial properties^13^. Honey is usually made by bees, most commonly the European honey bee, *Apis mellifera*, from the nectar of flowering plants. The honey types derived from different plants vary substantially in their antimicrobial activity^14–18^, which stems from multiple factors including high sugar content, low pH and the production of hydrogen peroxide via the bee-derived enzyme, glucose oxidase^19,20^. Certain honeys derived from the *Leptospermum* species of plants native to Australia and New Zealand (e.g. manuka honey) have an additional antimicrobial component called methylglyoxal, or MGO, which forms from the nectar-derived precursor compound, dihydroxyacetone (DHA), during the ripening of honey^21–23^. The level of MGO in *Leptospermum* honey has been positively correlated to the ‘non-peroxide activity’, referring to the antibacterial activity remaining in honey following neutralisation of hydrogen peroxide via the addition of catalase, in previous studies^21–23^. These honeys are the most commonly used for medical-grade honey products as their MGO-derived non-peroxide activity is not affected by catalase present in the body and they are available in the form of various sterile products licensed for use in wound care^24,25^.

The antimicrobial action of New Zealand manuka-based honeys have been demonstrated *in vitro* against a wide range of problematic bacterial pathogens, including those that can colonise the skin and wounds such as *Staphylococcus aureus* and *P. aeruginosa*^26^. Of particular note is that manuka honey is equally effective at inhibiting multi-drug resistant clinical isolates as it is against sensitive strains, indicating a broad spectrum of activity unlike that of other known antibiotics^27–29^. In addition, bacteria are unable to develop resistance to honey, even under conditions that rapidly induce resistance to common antibiotics^30,31^.

As well as inhibiting planktonic cell growth, honey has previously been demonstrated to have anti-biofilm activity *in vitro*. Manuka honey prevents the formation of biofilms by many problematic wound pathogens, including *Staphylococcus* and *Streptococcus* species, *Acinetobacter baumannii*, *Eschericia coli*, *Enterobacter cloacae* and *P. aeruginosa*, and it eradicates established biofilms^32–40^. However, the levels of reported anti-biofilm activity are not consistent among all studies (ranging from 12–50%), which is likely to be due to differences in the levels of the major antibacterial components in the honey. Although MGO has been shown to have inhibitory action against established *S. aureus* and *P. aeruginosa* biofilms previously^41^, it is not solely responsible for the anti-biofilm activity of manuka-type honeys highlighting the importance of additional components in the honey that modulate activity^38^.

Here we have assessed the anti-biofilm activity of four New Zealand honeys, including three manuka-based samples, and their key antibacterial components (i.e. MGO, hydrogen peroxide and sugar) against two *P. aeruginosa* strains of different biofilm-forming ability. The honey samples were characterised in terms of their geographical and floral source and the levels of the two major antibacterial components, MGO and hydrogen peroxide. We demonstrate that the honeys are active in both the prevention and eradication of *P. aeruginosa* biofilms, and that MGO is not the major driver of anti-biofilm activity of the manuka-type honeys. While the sugar solution (control) was demonstrated to be similarly effective in biofilm prevention and eradication, this does not negate the use of honey over sugar solutions in clinical practice as sugar alone is not as effective at similar concentrations to honey against other common wound pathogens^30,38,42–46^. This study also emphasises the importance of using well-characterised honeys in order to understand the antimicrobial and anti-biofilm activity and to choose the most appropriate honey for treating infected wounds.

## Methods

### Bacterial strains and growth conditions

The two laboratory reference strains of *P. aeruginosa* used in this study, PAO1 (ATCC 15692) and PA14 (UCBPP-PA14), were originally isolated from burn wounds^47,48^. Strains were grown in cation-adjusted Mueller-Hinton broth (CAMHB; Becton Dickinson Biosciences, USA) at 37 °C.

### Honey samples and control solutions

The four honey samples used in this study were all of New Zealand (NZ) origin and included three manuka-type honeys: monofloral manuka honey, Medihoney (a manuka-based medical-grade honey), and a manuka-kanuka blend, as well as a clover honey. All honey samples were supplied by Comvita NZ Ltd (Te Puke, New Zealand). Floral source, harvesting and geographic information, as well as the levels of methylglyoxal (MGO) and the MGO pre-cursor compound, di-hydroxyacetone (DHA) were supplied by Comvita NZ Ltd and are shown in Table 1. All honey samples were stored in the dark at 4 °C and were freshly diluted in CAMHB immediately before use in assays. All honey concentrations are expressed as % weight per volume (w/v).

**Table 1:** Harvesting, geographical and composition data for honey samples.

Honey type	Harvest period	Area	Floral source	Major antimicrobial components		
				MGO^a^	DHA^b^	H_2_O_2_^c^
Manuka	Spring 2010	Hokianga, Northland, NZ	*Leptospermum scoparium var. incanum*	958	4277	0.34
Medihoney	Spring 2010	Northland, NZ	*Leptospermum scoparium var. incanum* + *Kunzea ericoides*	776	883	0.31
Manuka-kanuka	Summer 2010/11	Hokianga, Northland, NZ	*Leptospermum scoparium var. incanum* + *Kunzea ericoides*	161	652	0.68
Clover	N/A	Balcutha, Otago, NZ	*Trifolium* spp.	<10	<20	0.11

^a^MGO (methylglyoxal) levels expressed as mg per kg of honey.

^b^DHA (dihydroxyacetone) levels expressed as mg per kg of honey. DHA exhibits no antimicrobial activity itself, but converts to the antimicrobial compound, MGO. DHA levels provide an estimate of the potential antimicrobial activity of *Leptospermum* honey.

^c^H_2_O_2_ (hydrogen peroxide) rate of production expressed as mmol/h in 1 ml of 10% w/v honey; measured in ten minute intervals over the course of 40 min.

^*^N/A: not applicable.

The following control solutions were also included in this study: (i) a sugar solution composed of glucose, fructose and sucrose (45 g, 38 g and 1 g, respectively) prepared in water (16 ml) and designed to mimic the concentration and composition of the main sugars in honey; (ii) an MGO solution prepared in CAMHB at concentrations similar to those present in the manuka-type honeys (i.e. 100 mg/kg, 700 mg/kg, and 900 mg/kg) to assess the effects of MGO alone; and (iii) MGO diluted (to the same concentrations as (ii)) in sugar solution to assess the combined effects of MGO and the main sugars of honey. MGO was sourced as a ~40% (w/w) solution in water (Sigma-Aldrich Co., MO, USA). Control solutions were stored and freshly diluted as per the honey samples above.

### Hydrogen peroxide assay

The levels of hydrogen peroxide (H_2_O_2_; another major antimicrobial component in honey) produced by the honey samples was determined using an Amplex Red hydrogen peroxide/peroxidase kit (Molecular Probes, Life Technologies Corp., Carlsbad, CA, USA) as previously described^49^, and are included in Table 1.

### Susceptibility of *P. aeruginosa* to honeys

The minimum inhibitory concentrations (MICs) of the honeys and sugar solution for *P. aeruginosa* were determined using the CLSI broth microdilution method^50^, with some modifications described below.

Overnight cultures of *P. aeruginosa* (2 ml, shaking at 250 rpm) were diluted to give a final cell density of 10^7^ CFU/ml in fresh CAMHB containing the appropriate test solution (honey or sugar). Honey stock solutions (50% w/v) were freshly prepared, and further diluted 2-fold serially in CAMHB to the required test concentrations ranging from 1–32%.

Media (CAMHB) alone was included as a growth (untreated) control. The assay was set up in 96-well microtitre plates (BD Falcon, NJ, USA), covered with AeraSeal (Excel Scientific, CA, USA) and incubated for 24 h at 37 °C in a humidified incubator (Thermo Fisher Scientific, USA). Cell growth was measured by optical density at 595 nm (OD_595_) in a plate reader (VersaMax, Molecular Devices, California, USA) and the MIC was defined as the lowest concentration at which the OD was ≤5% relative to the untreated control, indicating at least 95% inhibition of cell growth.

### Biofilm formation assays

The effect of the test solutions (honey and control solutions) on *P. aeruginosa* biofilm formation was determined using crystal violet static biofilm assays in microtitre plates as previously published^51^, with some modifications.

*P. aeruginosa* biofilms were prepared in CAMHB with several concentrations of honey, as described above. After 24 h incubation at 37 °C, the microtitre plates were washed three times in an automated plate washer (Bio-Tek, ELX405, Winooski, VT, USA) with phosphate buffered saline (PBS) to remove unattached cells. The plates were stained with 0.2% w/v crystal violet, incubated at room temperature for 1 h and excess crystal violet solution washed out using the same program above. The stain that was bound to the adherent biofilm biomass was resolubilised in acetic acid (33% w/v, 200 µl), transferred to a new microtitre plate and the OD_595_ measured. The minimum biofilm inhibitory concentration (MBIC) was determined as the lowest concentration at which the OD was ≤5% of that of the untreated control, indicating at least 95% inhibition of biofilm formation.

### Biofilm elimination assays

*P. aeruginosa* biofilms were first allowed to form in the wells of mictrotitre plates for 24 h at 37 °C as described above, but in CAMHB only (i.e. no treatment). The plates were then washed three times with PBS, and various concentrations (0%, 1%, 2%, 4%, 16%, and 32%) of the four honeys and control solutions were added to the established biofilms. The wells containing 0% treatment concentration were made up to volume using CAMHB. The plates were further incubated for 24 h at 37 °C, and the biofilm biomass was quantified using crystal violet as above.

### Bacterial cell viability in biofilms

The viability of cells within the *P. aeruginosa* biofilms following treatment with honey or control solutions (for 24 h, as above) was quantified by the BacTitre Glo Microbial Cell Viability Assay Kit (Promega, WI, USA), which measures ATP levels via a luminescence-based luciferase activity assay as an indicator of cell viability^52,53^. After treatment, bacterial biofilms were washed as described above, followed by incubation with the BacTitre Glo reagent in CAMHB for 10 min at 37 °C in the dark.

The assay was performed and validity checked against a standard curve as previously described for *Staphylococcus aureus*^38^, with the modification of using CAMHB for *P. aeruginosa* biofilms. Biofilms were produced and washed as above and cells within the biofilm dispersed using a small-probe sonicator (8 sec at 40% power; Sonics and materials VC-505) to enable quantification by direct enumeration. Bacterial colony forming units (CFUs) per well were calculated and plotted against the luminescent readings from the corresponding well to generate the standard curve (Supplementary Fig. S1). The lower detection limit of the BacTitre Glo assay was at the luminescence value of <1000, which is equivalent to 10^3^ CFU/ml.

### Visualising *P. aeruginosa* biofilms using confocal laser scanning microscopy (CLSM)

*P. aeruginosa* biofilms treated with 1%, 2%, 16%, and 32% of each of the four honeys or the sugar solution control (all prepared in CAMHB) were visualised using live/dead staining with Syto9 (Invitrogen, CA, USA) and propidium iodide (Becton Dickinson, NJ, USA) and imaged using confocal laser scanning microscopy, as previously described^38^. As higher concentrations of honey are used in the commercially available honey-based wound dressings, higher concentrations (64% and 80%) of the monofloral manuka honey and Medihoney samples were also tested.

### Susceptibility of *P. aeruginosa* cells recovered from treated biofilms to honeys

As it is known that bacteria are more likely to become resistant to antimicrobial compounds following exposure to sub-inhibitory concentrations^54,55^, the susceptibility of *P. aeruginosa* following exposure to sub-inhibitory concentrations of honey was determined using the MIC and MBIC methods described above. *P. aeruginosa* cells recovered from biofilms treated with sub-inhibitory concentrations (8%) of the manuka-type honeys were tested to determine their ability to grow and form biofilms in the presence of higher (previously inhibitory) concentrations of these honeys (16% and 32%).

### Statistical analyses

Statistical analyses to compare treatments (honeys and control solutions) were performed using GraphPad Prism (versions 5 and 6). Normal (Gaussian) distribution of data were checked using the D’Agostino-Pearson normality test (alpha = 0.05). Differences among honey samples and across control solutions were determined using One-Way ANOVA with Tukey Test, with statistical significance set at p < 0.05.

## Results

### Effect of honey on *P. aeruginosa* growth and biofilm formation and re-assessment of susceptibility following honey treatment

The effects of the four NZ honeys and control solutions on *P. aeruginosa* planktonic cell growth and biofilm formation were assessed. The two *P. aeruginosa* strains used in this study, PAO1 and PA14, had different biofilm forming abilities (Supplementary Fig. S2), and all honeys were effective at inhibiting the planktonic cell growth and biofilm formation of both strains. Planktonic growth of both *P. aeruginosa* strains was completely inhibited by 16% of the three manuka-type honeys and by 32% clover honey (Table 2). The sugar solution also inhibited PAO1 growth at 32%, but PA14 was not inhibited at any of the tested sugar concentrations (1–32%).

**Table 2:** Concentrations of honey required to inhibit *P. aeruginosa* cell growth and biofilm formation before and after exposure to honey.

Honey	Initial susceptibility^a^				Susceptibility after exposure to honey^b^			
	PAO1		PA14		PAO1		PA14	
	MIC	MBIC	MIC	MBIC	MIC	MBIC	MIC	MBIC
Manuka	16	16	16	8	32	32	32	32
Medihoney	16	32	16	16	32	32	32	32
Manuka-kanuka	16	16	16	16	32	32	32	32
Clover	32	32	32	16	N/A	N/A	N/A	N/A
Sugar solution	32	32	>32	32	N/A	N/A	N/A	N/A

All concentrations are expressed as percentage weight per volume (% w/v).

^a^Initial susceptibility of PAO1 and PA14 to honeys.

^b^Susceptibility of PAO1 and PA14 cells recovered from biofilms that had been treated with a sub-inhibitory concentration (8%) of honey.

N/A: not applicable as these honeys were not tested in resistance assays.

Biofilm formation was inhibited by the four tested honeys, and the sugar solution for both PAO1 and PA14. Generally, the MBICs were the same as the MICs, however the MBIC for PAO1 was 2-fold higher (32%) with Medihoney and for PA14 this was 2-fold lower (8%) with manuka honey (Table 2). The MBIC for clover honey and the sugar solution was 32%, with the following exceptions: the MBIC for PA14 with clover honey was 16% i.e. 2-fold less than the MIC; and the MBIC of the sugar solution was 32% while the MIC was >32%.

Table 2 also shows susceptibility testing of *P. aeruginosa* cells derived from the honey-treated biofilms. Generally the MICs and MBICs increased at least 2-fold.

**Figure 1:**
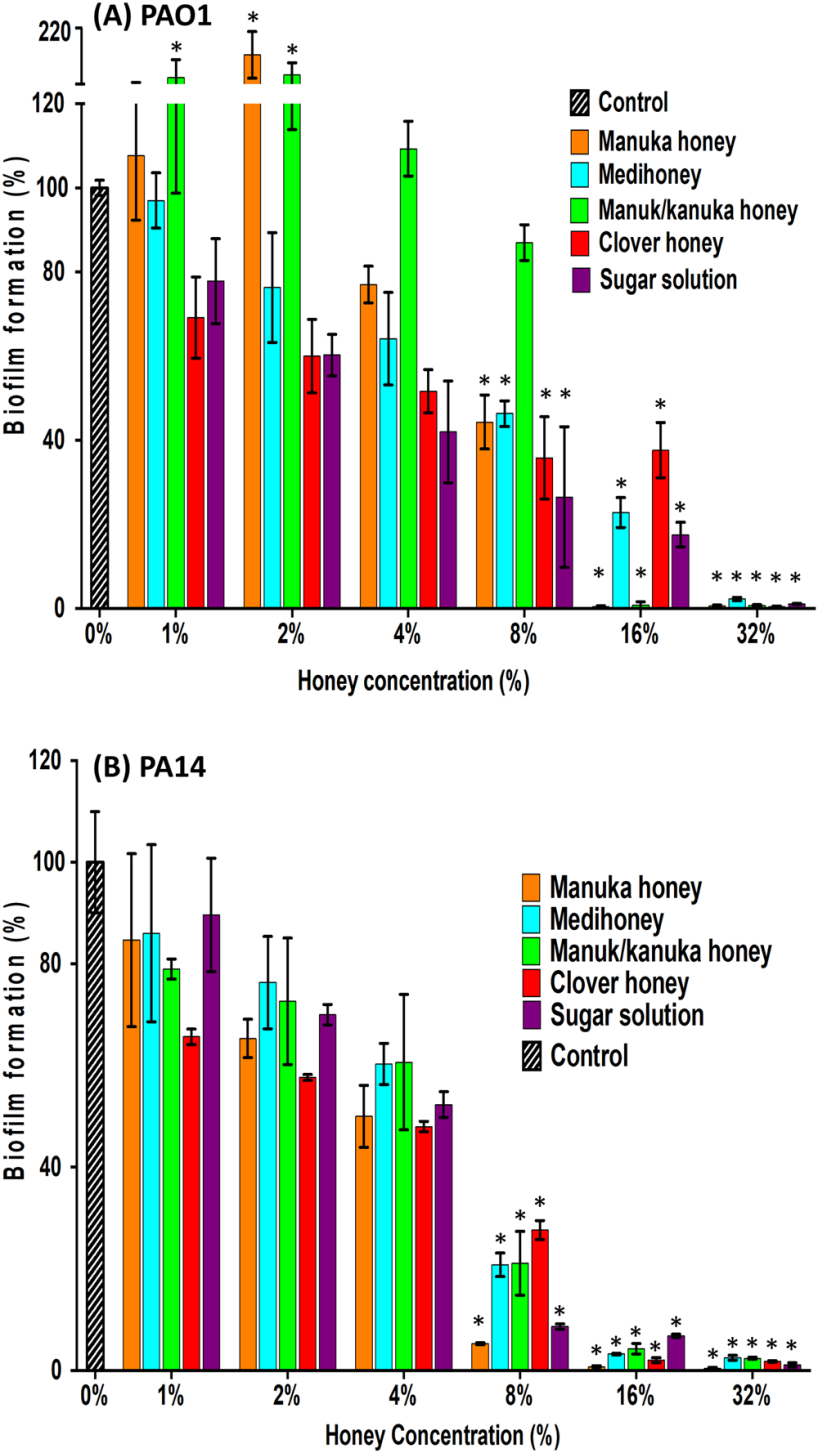
Effect of honey and sugar solution on *P. aeruginosa* biofilm formation. *P. aeruginosa* PAO1 (**A**) and PA14 (**B**) biofilms were allowed to form in the presence of four NZ honeys (manuka, Medihoney, manuka-kanuka, or clover) or a sugar solution. Biofilm formation was assessed using a static biofilm formation assay with crystal violet staining to quantify biomass. Biofilm formation is expressed as % relative to that produced by the untreated control (Control), which is set at 100%. Error bars represent ± SD of three biological samples, all performed in triplicate. *Indicates statistically significant difference (p < 0.05) relative to the untreated control (Control; 0%).

In some cases, low concentrations of manuka and manuka-kanuka honey enhanced biofilm formation (Fig. 1). Biofilm biomass of PAO1 was significantly enhanced (p < 0.05) by sub-inhibitory concentrations of manuka (2%) and manuka-kanuka (1% and 2%) honey. This effect, which has previously been seen with certain antibiotics, was not observed in strain PA14.

### Effect of honey on established *P. aeruginosa* biofilms

**Figure 2:**
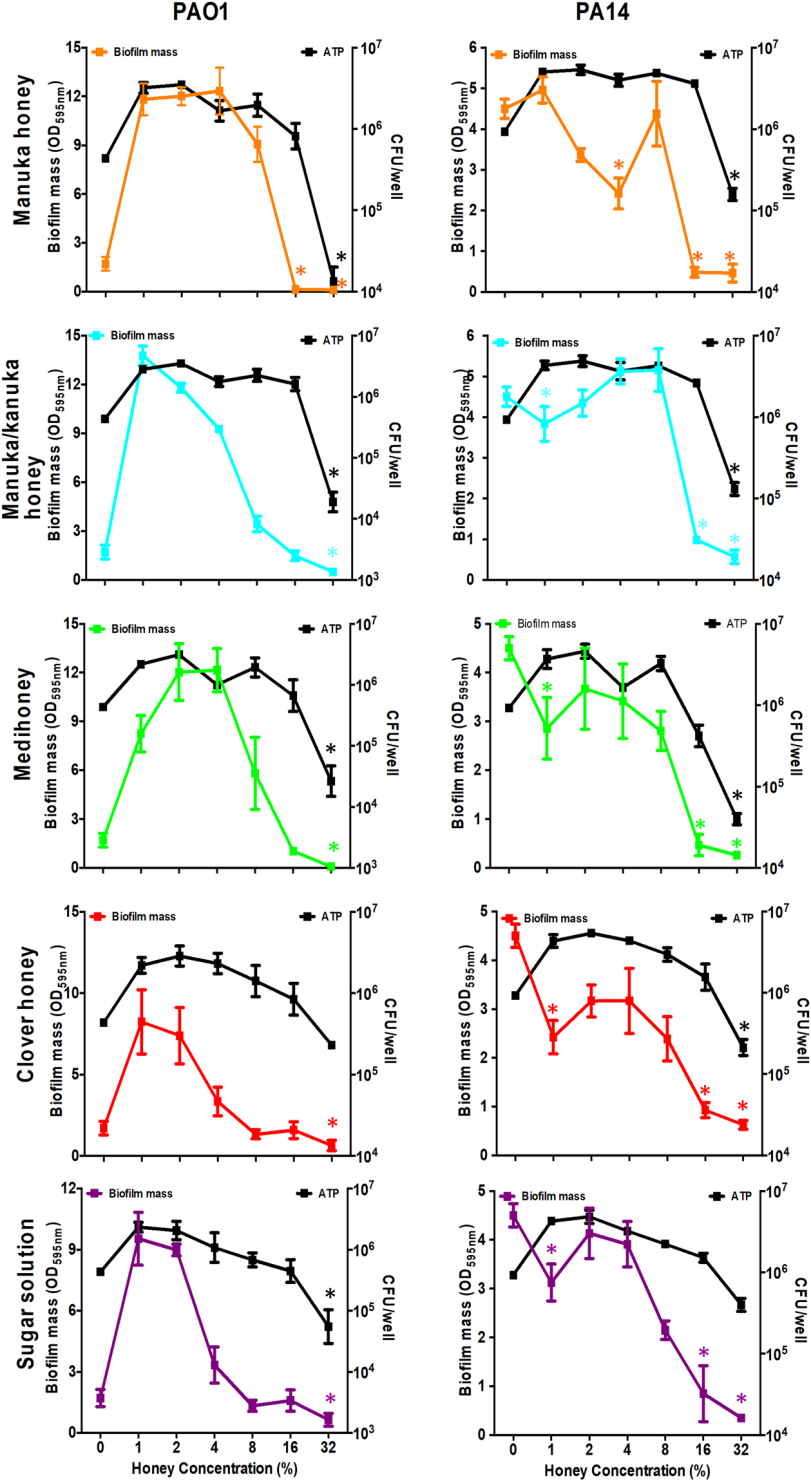
Effect of honey or sugar solution on established *P. aeruginosa* biofilms and on cell viability within the biofilms. Established *P. aeruginosa* PAO1 (left panels) and PA14 (right panels) biofilms were treated with four NZ honeys (manuka, Medihoney, manuka-kanuka, and clover), or a sugar solution. Biofilm biomass remaining post-treatment (coloured lines) was quantified using crystal violet staining and expressed as OD_595_ (left y-axis). The corresponding cell viability (black line) within remaining biofilms was assessed via ATP production using the BacTitre Glo Viability Kit and CFU/well values were determined from a previously established standard curve (right y-axis). Data represents mean values from three biological replicates, all performed in triplicate ± SD. Coloured (*) indicate statistically significant decrease (p < 0.05) in biofilm biomass and black (*) indicates statistically significant difference (p < 0.05) in ATP production, both relative to the control (at 0% honey concentration).

As bacterial biofilms are already established in chronic wounds, the ability of honey to eradicate pre-formed *P. aeruginosa* biofilms was investigated. In general, treatment with 16% or 32% honey resulted in a significant (p < 0.05) reduction in biofilm biomass (Fig. 2). The two strains of *P. aeruginosa* differed in their responses to the lower concentrations of honey and this may be attributed to their different biofilm forming abilities shown in Supplementary Fig. S2 and also indicated by the markedly different biomass values at the 0% honey concentration in Fig. 2. For PAO1, the biofilm biomass was significantly enhanced when sub-inhibitory concentrations (1–4%) of any of the honeys or the sugar solution was used (Fig. 2). Interestingly, the PA14 results showed that at very low levels (1%) of the manuka-kanuka honey, Medihoney, clover honey and the sugar control, biofilm biomass was generally inhibited, then augmented with higher levels of the treatments until it became inhibitory at the highest levels (Fig. 2). For PAO1 there was <40% reduction in biofilm mass following treatment with all honeys except manuka, while all four honeys and the sugar solution reduced the biofilms established by strain PA14 by ≥75% relative to the untreated control (Supplementary Table S1).

An ATP-based viability assay was used to approximate the number of viable cells remaining within the biofilm after treatment with the honeys or sugar solution. In general, cell viability decreased significantly for all treatments at 32% (Fig. 2 and Supplementary Table S1). While 16% reduced the established biofilm biomass of both PAO1 and PA14, cell viability significantly increased (p < 0.05) or there was no change compared to the untreated control. Additionally, sub-inhibitory concentrations (1% and 2%) of the different honey or sugar solution treatments enhanced PA14 cell viability within the established biofilms (p < 0.05) but not the biofilm biomass (p > 0.05) (Fig. 2).

**Figure 3:**
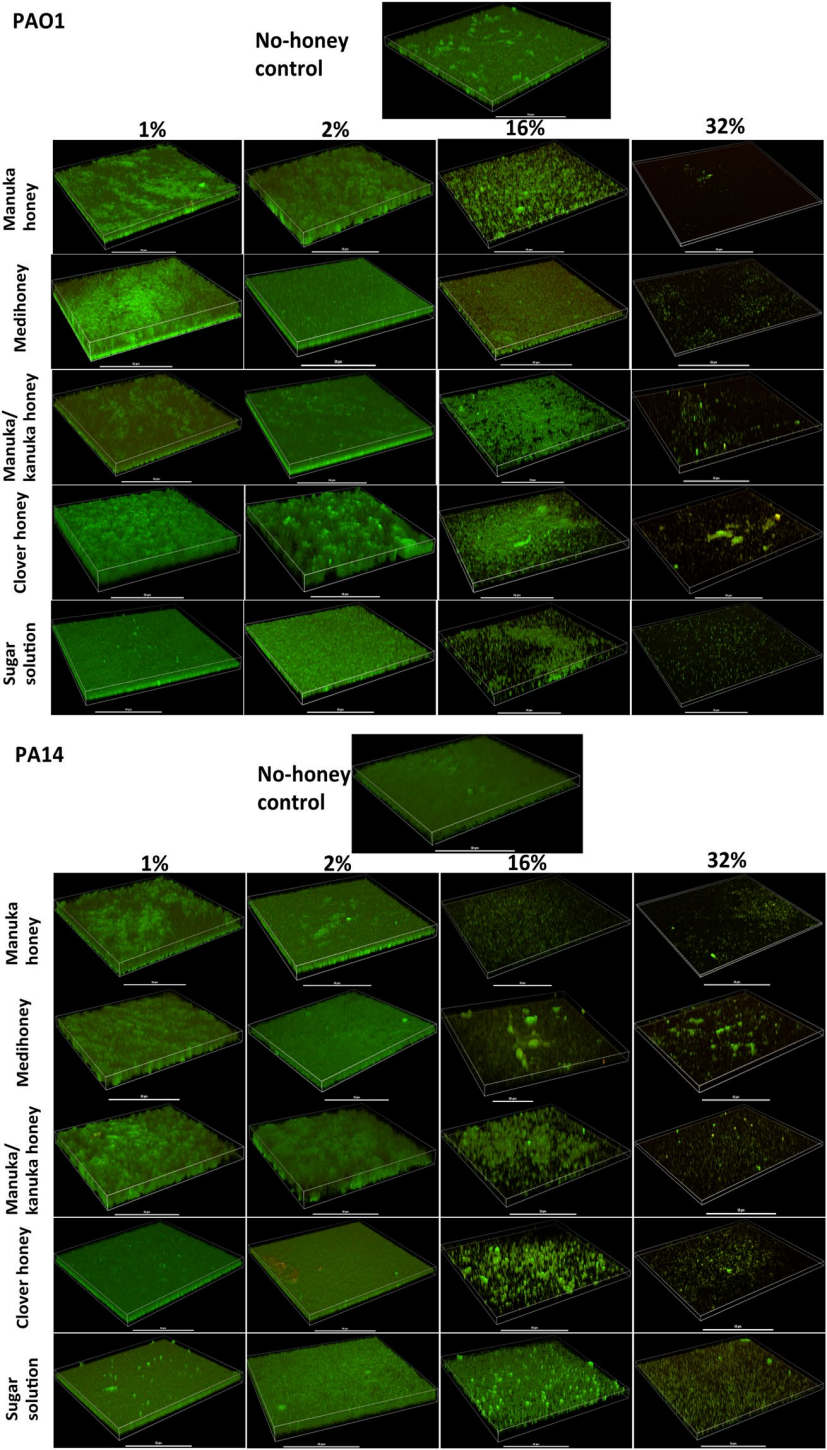
Visualisation of established *P. aeruginosa* biofilms treated with different honeys. 3-D images produced by confocal laser scanning microscopy of established *P. aeruginosa* PAO1 and PA14 biofilms, following treatment with sub-inhibitory (1 and 2%) and inhibitory (16 and 32%) concentrations of NZ honeys (manuka, Medihoney, manuka-kanuka, or clover) or control sugar solution. Biofilms were stained with Syto9 (green = viable cells) and propidium iodine (red = dead cells). Scale bar represents 50 µm.

The effect of the NZ honeys and the control sugar solution at sub-inhibitory (1% and 2%) and inhibitory (16% and 32%) concentrations on established biofilms was further examined using confocal laser scanning microscropy (CLSM) to visualise the biofilms and assess viability within the biofilm (Fig. 3). At concentrations of 16%, the total amount of cells was visibly decreased and more dead cells (red; stained with propidium iodide) were observed (Fig. 3). At 32%, all treatments caused a substantial visual reduction in the amount of live cell lawn (green; stained with Syto9), with fewer live or attached cells at the imaged surface area. These results are consistent with the observations in the biofilm eradication assay, where the tested honeys and sugar solution significantly decreased the established *P. aeruginosa* biofilm biomass (Fig. 2 and Supplementary Table S1).

**Figure 4:**
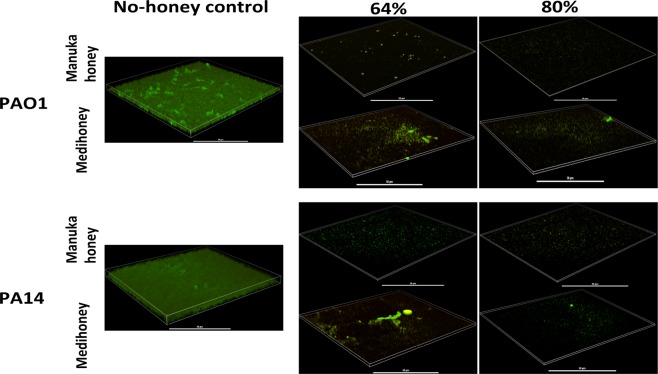
Effects of high concentrations of manuka and Medihoney on established *P. aeruginosa* biofilms. 3-D images produced by confocal laser scanning microscopy of established *P. aeruginosa* PAO1 and PA14 biofilms, following treatment with high concentrations (64 and 80%) of manuka honey and Medihoney. Biofilms were stained with Syto9 (green = viable cells) and propidium iodine (red = dead cells). Scale bar represents 50 µm.

Higher concentrations (64% and 80%) of the two medical-grade honeys (manuka honey and Medihoney) were also tested on established *P. aeruginosa* biofilms (Fig. 4) to better reflect the clinical situation, where these honeys are used at high concentrations as an antibacterial wound treatment. CLSM imaging showed that these medical-grade honeys were effective at reducing established *P. aeruginosa* biofilms, with very few cells remaining on the imaged surface relative to the untreated control (Fig. 4). Visually, there were also fewer fluorescently stained cells remaining attached on the imaged surfaces, compared to 32% of the same honey treatment (Figs. 3 and 4).

### Effect of MGO on *P. aeruginosa* biofilm formation and eradication

Methylglyoxal (MGO) makes a substantial contribution to the activity of manuka and related honeys, and has previously been demonstrated to be effective at both inhibiting biofilm formation and eradicating established *P. aeruginosa* biofilms^41^. To determine the contribution of MGO to the biofilm prevention and eradication activity of the manuka-type honeys in this study, MGO was tested both with and without the addition of the sugar solution at concentrations representative of those in each of the manuka-type honeys (Table 1).

**Figure 5:**
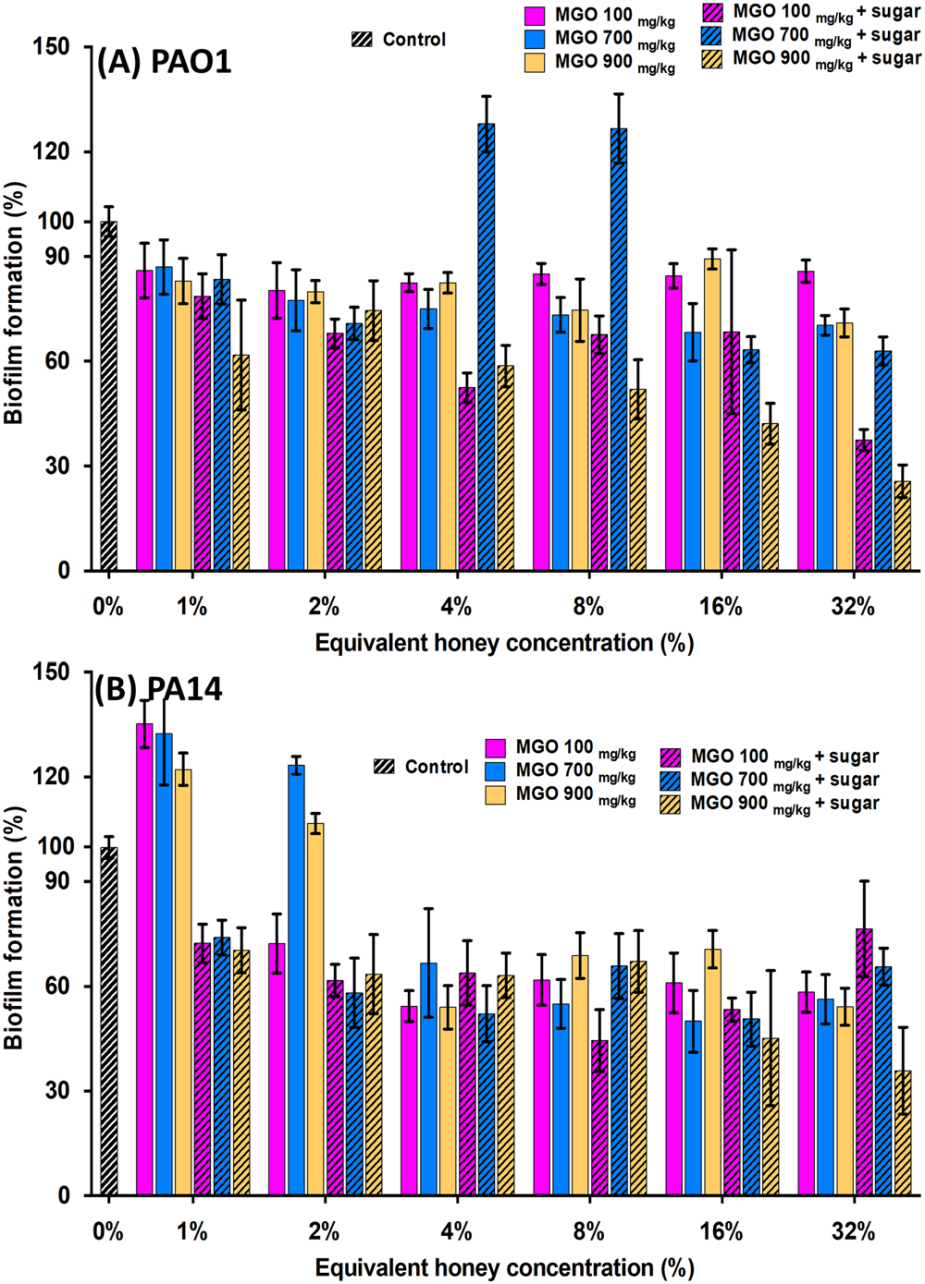
Effect of MGO on biofilm formation by *P. aeruginosa*. Biofilm formation by *P. aeruginosa* PAO1 (**A**) and PA14 (**B**) in the presence of MGO and MGO plus sugar solution. MGO concentrations correspond to those present in the manuka-type honeys: 100 mg/kg as in manuka-kanuka honey, 700 mg/kg as in Medihoney, and 900 mg/kg as in manuka honey. Biofilm formation is expressed as a percentage relative to the untreated control, which is set at 100%. Results presented are mean values from three biological replicates, all performed in triplicate ± SD.

Some reduction in biofilm formation was observed using MGO alone or in combination with sugar (Fig. 5), however, the MGO solutions did not reproduce the inhibitory effects observed with the manuka-type honeys (Fig. 1). The biggest reduction in biofilm formation was observed when MGO solution, with or without sugar, was used at the concentration equivalent to manuka honey, i.e. 900 mg/kg (Fig. 5). Biofilm formation was reduced by up to 50% when MGO alone was used at a concentration of 16% honey equivalence (Fig. 5). MGO with sugar used at a concentration of 32% honey equivalence resulted in a 75% reduction in biofilm formation (Fig. 5). In contrast, >95% inhibition was seen with the corresponding honey at the equivalent MGO concentrations (Fig. 1). The addition of MGO to the sugar solution also appeared to counteract the inhibitory effect observed for the sugar solution alone for biofilm formation (Figs. 1 and 5), and some sub-inhibitory concentrations of MGO (alone or in combination with sugar solution) enhanced biofilm formation.

**Figure 6:**
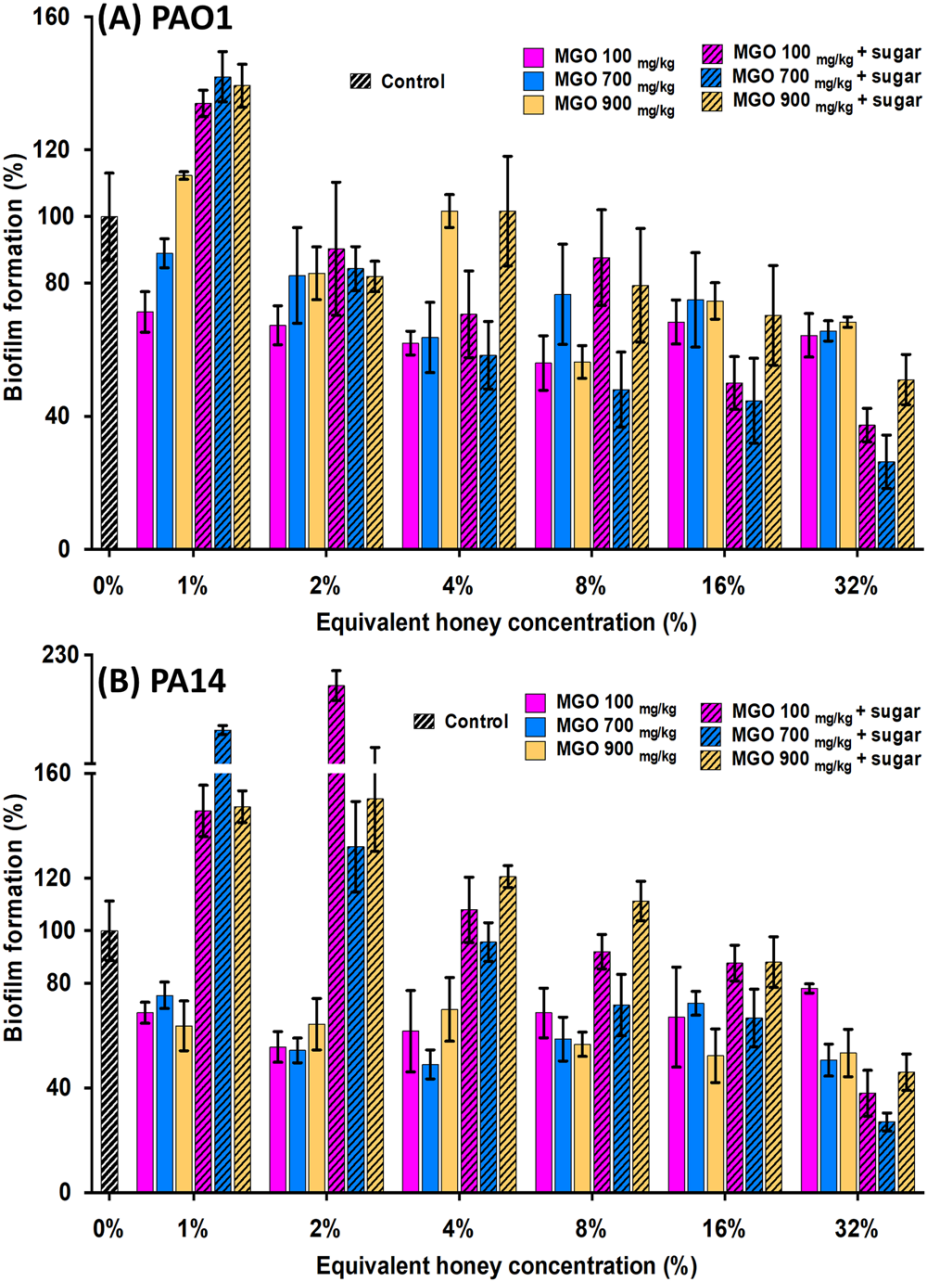
Effect of MGO on established *P. aeruginosa* biofilms. *P. aeruginosa* PAO1 (**A**) and PA14 (**B**) biofilms treated with MGO, with and without sugar solution. MGO concentrations used correspond to those in the manuka-type honeys: 100 mg/kg as in manuka-kanuka honey, 700 mg/kg as in Medihoney^M^, and 900 mg/kg as in manuka honey. Biofilm formation is expressed as a percentage relative to the untreated control, which is set at 100%. Results presented are mean values from three biological replicates, all performed in triplicate ± SD.

As with the biofilm inhibition assays, treatment with MGO at concentrations similar to those present in the manuka-type honeys did not reduce the biofilm biomass to the same degree as the corresponding honey (Figs. 6 and 2). While there was some reduction in biofilm mass by 32% MGO alone or in combination with sugar (Fig. 6), this was markedly less than that of the manuka-type honeys at this concentration (Fig. 2).

## Discussion

Chronic wounds harbour bacterial populations that commonly exist as biofilms^41^, which are known to be far more tolerant to antibiotics than planktonic bacteria^56,57^. Ideally, effective treatments of chronic wounds should have broad-spectrum antimicrobial activity effective against multi-drug resistant wound pathogens, as well as the ability to reduce or eradicate existing biofilms in the wound, while simultaneously preventing the formation of new biofilms. The need for new chronic wound treatments coupled with the rise in antibiotic resistance has prompted renewed interest in complex, natural products with antimicrobial activity–like honey, and the use of manuka-type honeys for the treatment of chronic wounds is especially promising. Here we show that manuka-type honeys have the ability to prevent and eradicate biofilms formed by the common wound pathogen, *P. aeruginosa*, notorious for being recalcitrant to conventional treatments.

The manuka-type honeys tested inhibited *P. aeruginosa* planktonic cell growth and the formation of biofilms, and also eliminated established biofilms at concentrations that could be maintained in wound dressing (8–32%; Table 2 and Figs. 1–4). This is in general agreement with the literature as several studies have reported on the effectiveness of manuka-type honeys against *P. aeruginosa*^33,34^. However, the amount of honey required to inhibit *P. aeruginosa* growth and biofilm formation varies between these studies, which report concentrations of honey between 12–50% as being inhibitory. In comparison, the growth and biofilm inhibitory concentration against *P. aeruginosa* in our study was generally at 16% honey. These differences are likely to be due to the different strains of *P. aeruginosa* tested in the studies and their relative biofilm-forming abilities as this is known to vary among strains. Differences between specific manuka-type honeys used e.g. the age, floral source, or processing and storage conditions of the honey samples can also affect the inhibitory action observed^16,58,59^. Most studies examining the antimicrobial effects of honey do not thoroughly characterise the honeys tested, despite the fact that the chemical composition and specifically the concentrations of the major antibacterial components (hydrogen peroxide, antimicrobial peptides, phenolics, or MGO) vary from one honey type to another and significantly affect the antimicrobial activity^20–22,60^. This highlights the importance of characterising the honey types tested, for example, by determining the levels of their key antibacterial components for ease of comparison between studies and for determining the most appropriate honeys for use in the clinic. However, fully characterising honey can be very difficult due to its complex nature, and even when done individual components, or combinations of these, may not necessarily align to inhibition very well.

MGO is believed to be one of the major antibacterial and anti-biofilm components of manuka honey, with demonstrated inhibitory effects against a range of bacteria^41^. In previous studies, the anti-biofilm activity of manuka-type honey against *S. aureus* and *P. aeruginosa* has been largely attributed to MGO^22,41,61^. Under the conditions used here however, MGO treatment at concentrations broadly corresponding to those in the manuka-type honeys, tested alone or in the presence of sugar, did not induce similar biofilm inhibitory or eliminatory effects (Figs. 5 and 6). This suggests that MGO is not the main driver of the anti-biofilm effects of manuka-type honey. Indeed our results indicate that MGO contributes very little to the anti-*P. aeruginosa* biofilm activity (Figs. 5 and 6). Although our results do not agree with previous studies, this is likely to be because of the different *P. aeruginosa* strains used and more directly related to the differences in the MGO concentrations tested. Previous studies showing successful *P. aeruginosa* biofilm inhibition by MGO alone have generally used much higher concentrations of MGO (between 1800–7300 mg/kg)^41^ than used here (288 mg/kg, equivalent to 32% manuka honey). The high concentrations of MGO used in other studies are well above the highest reported levels of MGO in manuka-type honeys (~1100 mg/kg)^23,62^ and may have toxic effects on host cells^63^. Our findings suggest that *P. aeruginosa* is markedly more tolerant to MGO than other common wound pathogens, such as *S. aureus*^49^, consistent with previous reports^41^. This is likely to be due to the ability of *P. aeruginosa* to detoxify MGO in several ways via the glyoxylase system (composed of glyoxalase I and II) and *P. aeruginosa* is known to have three fully functional glyoxylase I homologues, whereas most bacteria contain one glyoxylase I gene^64^.

Under the conditions tested here, sugar alone was generally almost as effective as the honey treatments (Figs. 1 and 2). However, since wound infections are polymicrobial, and we and others have previously shown that sugar is not as effective as manuka-type honeys against other common wound pathogens such as *S. aureus*^38^, sugar is not likely to be an equal or better option for chronic wound treatment, especially when the added wound healing and anti-inflammatory properties of honey^13^ are taken into account. The contribution of the sugar component of honey to the anti-biofilm effects observed here may be due to high osmolarity or to a nutrient effect, such as through changes to central carbon metabolism. Sugar in honey has been linked to disrupted quorum sensing in *P. aeruginosa*^65^, and high concentrations of fructose in honey or sugar solutions have been found to prevent *P. aeruginosa* biofilm formation^66^. It is also possible that *P. aeruginosa* is sensitive to the sugar solution due to its deficit in sugar transporters^67^. Adding osmotic pressure (e.g. via extra salt or sugar) is known to affect the swarming ability of bacteria and defects in swarming coincide with deficiencies in biofilm formation^68^. So, we tested the effect of the honeys and sugar solution on the swarming ability of *P. aeruginosa* to determine whether the anti-biofilm effect could be explained via this mechanism, however our results did not indicate this to be the case (Supplementary Table S2). While all the tested honeys and the sugar solution did affect the swarm colony size in both PA14 and PAO1, the manuka-type honeys showed marked reduction of the swarm size when used at a concentration of 8%, and colony inhibition at 16%, indicating that there are additional components in these honeys contributing to the overall anti-biofilm activity reported here. Future studies investigating how manuka-type honeys affect other mechanisms involved in *P. aeruginosa* biofilm formation (e.g. swimming motility or quorum sensing), may help to identify key components in honey responsible for its anti-biofilm action. These components could be integrated into wound dressings to test their direct biofilm inhibitory action, as has been done previously using the amino acid tryptophan^69^, which may provide better mechanistic insights.

While low concentrations (up to 32%) of any of the four honeys or the sugar solution were able to significantly reduce established *P. aeruginosa* biofilm biomass, these could not completely remove established biofilm (Fig. 3). In clinical practice, the honey concentration in honey-based wound dressings and gels is close to 100%, although there can be some dilution of the honey by wound exudate^70^. When the two medical-grade manuka-type honeys were tested at concentrations that better represent the concentrations used in clinical practice (64% and 80%), complete removal of established *P. aeruginosa* biofilms was observed (Fig. 4). Although *in vitro* studies can only provide insight to the *in vivo* situation, these results warrant further exploration of honey wound dressings for the treatment of infected chronic wounds.

Honey is an attractive substitute for topical antibiotics not only due to its broad-spectrum antimicrobial activity, but also because previous studies show that a range of bacteria capable of colonising the skin and wounds, including *P. aeruginosa*, *S. aureus*, *E. coli* and *A. calcoaceticus* do not develop resistance to honey, including medical-grade manuka honey^30,31^. This low propensity for resistance is likely to be due to the complexity of honey, which acts in a multifactorial way to target cells via several antibacterial compounds^30^. However, honey resistance has only been explored in planktonic cells and not within biofilm cells. Treatment of biofilm-associated chronic wounds often requires continued disruption of biofilms, where multiple doses of antibacterial agents over time are needed that may eventually induce resistance^71^. Here we tested biofilm cells recovered from sub-inhibitory manuka-type honey treatments for the development of resistance. These biofilm-recovered cells showed a 2-fold increase in MIC and MBIC (Table 1), suggesting that *P. aeruginosa* may acquire tolerance to honey treatment or that persister *P. aeruginosa* cells may develop during continued exposure to honey^72^. Further work is required to determine whether this decreased susceptibility is due to reversible, temporary tolerance or is an acquired state of honey resistance.

This study is the first to test a suite of well-characterised New Zealand honeys, and their key antibacterial components (sugar and MGO) against two *P. aeruginosa* strains with different biofilm forming abilities using a range of *in vitro* assays and fluorescent microscopy. We demonstrate that: (1) honey present at relatively low concentrations (up to 32%) compared to those used in honey-based wound dressings (~80–100%) inhibited the ability of *P. aeruginosa* to form biofilms and significantly reduced established biofilms; (2) the anti-biofilm effect of the manuka-type honeys was largely (but not wholly) driven by the sugar component and not MGO as previously suggested; (3) cells recovered from biofilms treated with sub-inhibitory concentrations of honey had slightly reduced susceptibility to honey; and (4) manuka-type honeys used at clinically obtainable concentrations (64% and 80%) completely eradicated established *P. aeruginosa* biofilms. Taken together, our results show that when used at appropriate concentrations, wound dressings saturated with manuka-based honey are promising effective treatments for infected chronic wounds, including those containing *P. aeruginosa* biofilms.

## Supplementary information

Supplementary Material

## Data Availability

All data generated or analysed during this study are included in this published article (and its Supplementary Information files).
