# Supplementary material for: Honey can inhibit and eliminate biofilms produced by Pseudomonas aeruginosa
Source: Sci Rep. 2019 Dec 3;9:18160. doi: 10.1038/s41598-019-54576-2 (PMC6890799; doi:10.1038/s41598-019-54576-2)
Supplement: Supplementary file 1 — Supplementary Material [file 41598_2019_54576_MOESM1_ESM.docx]

# Supplementary Material

Honey can inhibit and eliminate biofilms produced by *Pseudomonas aeruginosa*

Jing Lu^1^, Nural Cokcetin^1^, Catherine M Burke^1^, Lynne Turnbull^1^, Michael Liu^1^, Dee A Carter^2^, Cynthia B Whitchurch^1^ and Elizabeth J Harry^1*^

^1^ The ithree institute, University of Technology Sydney, Ultimo, NSW 2007, Australia

^2^ School of Life and Environmental Sciences, University of Sydney, Sydney, NSW 2006, Australia

* Correspondence: [liz.harry@uts.edu.au](mailto:liz.harry@uts.edu.au)


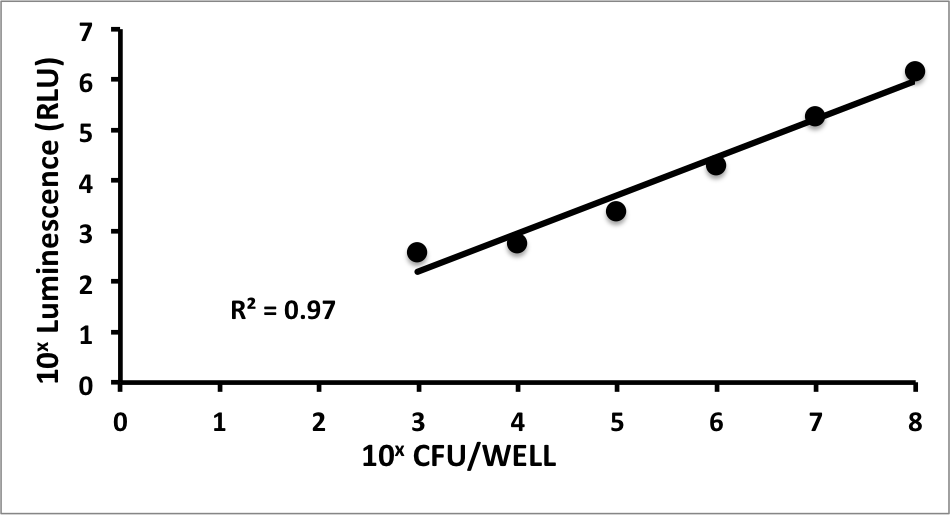


**Figure S1. Relationship between levels of intracellular ATP to colony forming units (CFU) in *P. aeruginosa* biofilms.**

Static biofilms of *P. aeruginosa* were allowed to form in the wells of a microtitre plate for 48 h (with media replenishment at 24 h). Biofilms produced were washed as described in the paper, and cells within the biofilm dispersed using a small-probe sonicator (Sonics and Materials VC-505) to enable quantification by direct enumeration. The sonicator microtip was inserted into each well and sonicated for 8 sec at 40% power. The viable count (CFU) of the recovered cells for each well was enumerated using a microdilution plate method.

Intracellular ATP levels were measured by the BacTitre Glo Viability Kit. The intracellular levels of ATP measured via luminescent signal were plotted as a function of CFU.

**Figure S2. Biofilm forming ability of *P. aeruginosa* strains used in this study.**

The ability of the two tested *P. aeruginosa* strains to form biofilms was determined by quantifying biofilm adherence over a 48 h period. A static biofilm formation assay was used to determine biofilm adherence at 24 h (A) and then after a further 24 h incubation (total of 48 h) with replenished fresh media (B). Biofilm adherence was quantified by using crystal violet staining (0.2 % solution) and measuring optical density (OD) at 595 nm. Error bars represent ± SD of three biological samples, all performed in triplicate. *** represents p < 0.01, determined by one-way ANOVA with Tukey test, following confirmation of data normality using the D’Agnostino-Pearson test.

**Table S1. Effect of honey and sugar solution at 16% and 32% on *P. aeruginosa* biofilm biomass and cell viability.**

|  | **PAO1** | | | | **PA14** | | | |
| --- | --- | --- | --- | --- | --- | --- | --- | --- |
|  | **16%** | | **32%** | | **16%** | | **32%** | |
| **Honey** | **Biofilm biomass** | **Cell viability** | **Biofilm biomass** | **Cell viability** | **Biofilm biomass** | **Cell viability** | **Biofilm biomass** | **Cell viability** |
| Manuka | 9% | 187% | 6% | 32% | 12% | 389% | 12% | 17% |
| Medihoney | 87% | 372% | 29% | 4% | 25% | 283% | 14% | 14% |
| Manuka-kanuka | 61% | 153% | 5% | 6% | 25% | 283% | 14% | 14% |
| Clover | 93% | 194% | 38% | 53% | 23% | 168% | 16% | 23% |
| Sugar solution | 93% | 103% | 38% | 13% | 21% | 163% | 9% | 43% |

Biofilm biomass and cell viability expressed as a percentage relative to the untreated control, which is set at 100 %.

Biofilm biomass remaining post honey treatment was quantified using crystal violet staining, and corresponding cell viability within the remaining biofilms was determined via ATP production using the BacTitre Glo Viability Kit.

**Table S2. Swarm colony size of *P. aeruginosa* in the presence and absence of honey or sugar**

|  | **Concentration of honey or sugar in plate (v/v)** | **PAO1 diammeter  (mm)** | **PA14 diammeter  (mm)** |
| --- | --- | --- | --- |
| **Manuka** | 4% | 64.2 ± 1.5 | 69.4 ± 0 |
|  | 8% | 34.9 ± 5.7 | 57.4 ± 5.7 |
|  | 16% | No growth | No growth |
| **Medihoney** | 4% | 69.6 ± 1.6 | 64.3 ± 0.5 |
|  | 8% | 33.3 ± 8.9 | 60.7 ± 1.4 |
|  | 16% | No growth | No growth |
| **Manuka-kanuka** | 4% | 53.5 ± 0.7 | 64.1 ± 2.5 |
|  | 8% | 22.3 ± 0.9 | 48.2 ± 1.7 |
|  | 16% | 8.8 ± 0.5 | No growth |
| **Clover** | 4% | 80.2 ± 0 | 79.8 ± 1.9 |
|  | 8% | 78.6 ± 1 | 81.4 ± 0.1 |
|  | 16% | 30.8 ± 4 | 28.7 ± 7.5 |
| **Sugar solution** | 4% | 79.1 ± 0.2 | 65.9 ± 3.5 |
|  | 8% | 54.5 ± 1.9 | 61.6 ± 0.3 |
|  | 16% | 26.3 ± 1 | 19.3 ± 1.3 |
| **Wildtype** | No added honey or sugar | 68.7 ± 1.1 | 71 ± 0.4 |

Results expressed as diameter ± SD of the swarm colonies. Diameter measured horizontally and vertically, and averaged for each experiment. Wildtype (no honey or sugar; control) experiments n=3; honey and sugar treated experiments n=2.

Swarming experiments were performed as previously described ^1^*,* with some modifications. Briefly, double-strength swarming agar plates were prepared by dissolving 6.4 g nutrient broth (Oxoid) and 4g Bacto-Agar (BD Biosciences) in 400 ml distilled water. After autoclaving, 10 ml of filter sterilised 40 % D-Glucose (Sigma Aldrich) prepared in distilled water was added to the agar to make up to a total volume of 400 ml. Honey and sugar solutions were prepared to double the required test concentrations (8, 16, 32% w/v) in sterile distilled water, and added 1:1 to the agar and pipetted into petri dishes (25 ml per plate). The final concentrations of honey or sugar in the swarming plates was equivalent to 4, 8, or 16 % v/v. The plates were dried in an aseptic spot for 40 min, and inoculated in the centre with 5 µl of an *P. aeruginosa* overnight culture (grown in Luria Bertoni broth; Oxoid), and incubated at 37 °C for 18 hours. The swarm colony diameter was measured horizontally and vertically, and averaged per plate.

1 Shrout, J. D. *et al.* The impact of quorum sensing and swarming motility on Pseudomonas aeruginosa biofilm formation is nutritionally conditional. *Mol Microbiol* **62**, 1264-1277, doi:10.1111/j.1365-2958.2006.05421.x (2006).
